# Highly efficient and rapid removal of Congo red dye from textile wastewater using facile synthesized Mg/Ni/Al layered double hydroxide

**DOI:** 10.1038/s41598-024-84604-9

**Published:** 2025-01-16

**Authors:** Eslam A. Mohamed, Hend M. Ahmed, Amal A. Altalhi, Hamdan A. S. Al-Shamiri, Nabel A. Negm

**Affiliations:** 1 Egyptian Petroleum Research Institute, Nasr City, Cairo Egypt; 2 Department of allied health professions, Faculty of Medical and Health Sciences, Liwa College, Abu Dhabi, UAE; 3 Department of Chemistry, College of Science, Taif University, P.O. Box 11099, Taif, 21944 Saudi Arabia; 4 Department of Physics, College of Science, University of Bisha, PO Box 551, Bisha, 61922 Saudi Arabia

**Keywords:** Layered double hydroxide, Dye uptake, Freundlich, Pseudo-second-order, Environmental chemistry, Environmental chemistry

## Abstract

Layered double hydroxides (LDH) are compounds with unique structures of hydroxide functional groups on their surfaces, and they have the proper arrangement of divalent and trivalent cations to adjust their unique catalytic actions. LDH was synthesized utilizing the co-precipitation technique and was thermally treated at 300 °C. The prepared compounds were chemically and structurally elucidated using FT-IR, XRD, SEM, BET, TG-DTA, and XPS characterization. We found that the thermal treatment of the prepared magnesium/nickel-LDH resulted in dehydration and dehydroxylation in its chemical structure. The crystallinity, the surface area, and the pore volume of the formed meso- and micropores were improved considerably after the thermal treatment. The efficiency of the uptake process was increased from 84 to 97% after the thermal treatment process, and the adsorption process tracked the Freundlich adsorption isotherm and pseudo-second-order kinetic model. The kinetics indicated the occurrence of three stages, and the diffusion of dye molecules into the pores was the rate-determining step. Different real water sample treatments showed the applicability of the thermally treated Mg/Ni/Al-LDH in the treatment process under optimized conditions. The presented mechanism of the uptake process using the prepared compounds comprises several interactions between the dye molecules and the thermally treated Mg/Ni/Al-LDH. The study presented the new application for Mg/Ni/Al-LDH in the as-prepared and thermally treated forms to uptake Congo-red (CR) dye from textile effluents.

## Introduction

LDHs are a class of compounds with unique structures that comprise the attendance of hydroxide moieties on their surfaces. The proper arrangement of divalent and trivalent cations in the LDHs chemical assembly adjusts their unique catalytic actions^1,2^. The supreme aspects considered through the creation of the LDH compounds are the types of metal cations, the alkalinity degree of the medium, the temperature, and the time required for the preparation progression, in addition to the preparation technique^3^. The most usable technique applied for the preparation of LDHs is the co-precipitating technique^4,5^. The co-precipitating technique permits outstandingly modification of the assembly of the LDHs by adjusting the molarity of the M(II)/M(III) ratio, the variety of negative inter-layer ions, the time of the process, and the alkalinity of the medium^6,7^. The most proper alkalinity of the medium is 9–10, higher pH causes leaching of divalent or trivalent ions, while low pH leads to incomplete precipitation of these ions in the LDHs arrangement. Urea is used in this protocol instead of NaOH and NaHCO_3_ to precipitate the lamellar LDHs by the gradual rise in the medium alkalinity during its decomposition by heat at 80–90 °C to attain the maximum alkalinity at 9–10^8^. Another protocol applied for the construction of LDHs is the sol/gel protocol, which allows cost, time, and energy-effective processes and attains highly pure products^9^. This protocol simply changed the reactants and time by replacement/addition of reactants. Before the balancing of the preparation conditions and components’ molar ratios, improving the pore and surface characteristics of the LDHs can be attained by their thermal treatment at a heating range from 150 °C to 750 °C in a process known as calcination^10^. LDHs are found in principal applications in environmental applications including adsorption of ionic (ions) and nonionic (organic dyes and pharmaceuticals) pollutants from wastewater. These threats are dramatically increased due to the draining of wastewater from industrial and agricultural sectors in rivers. The uptake of pollutants from the aquatic system using LDHs is a promising alternative for the adsorbents used including clays and carbonaceous derivatives^11,12^. Altered forms of LDHs successfully up-took atmospheric carbon and nitrogen dioxides due to their low cost, fast kinetic, high capacitance uptake, and superior thermal and chemical stabilities during the uptake cycles^13,14^. The adsorption capacities of CO_2_ ranged from 0.7 to 1.5 mmol.g^−1^^15^, and 0.602–0.651 mmol.g^−1^ in the case of NO_x_^16^.

CR harmfully affects living organisms and humans leading to fatal ailments and being toxic, carcinogenic, and mutagenic at high doses. CR is an anionic dye (azo-dye type) in its sodium salt form. It disturbs the reproductive and respiratory systems, skin, and eyes, and results in an allergic effect and even cancer tumors. CR is one of the azo dyes utilized in textile, paper, printing, and rubber productions and is mostly used as a dye in the textile industry^17,18^.

Ineffective textile dying procedures discharge 15–50% of the azo dyes that are not bonded to fibers and textiles into wastewater^19^. These types of effluents in wastewater have high toxicity for all types of organisms^20–23^. Adsorption using activated carbon, Sustainable biochar^24^, nano-filtration, and catalytic photodegradation were the main protocols for the exclusion of different dye grades from wastewater^25,26^. Concerning LDHs usage, they are characterized by low cost and highly regenerative ability during the uptake of CR dye operation^27,28^. In this study, magnesium/nickel/aluminum-LDH was prepared using the co-precipitation method and was functionalized with thermal treatment at 300 °C as innovative adsorbents with a distinct structural and compositional design that improves their adsorption ability for Congo red. Our improved synthesis approach produces well-crystalline LDH particles with a specified layered structure and an abundance of active sites. The LDH synergistic effect of magnesium, nickel, and aluminum cations promotes strong electrostatic interactions and π-π stacking with Congo red molecules, resulting in outstanding adsorption ability. The presented adsorbents remove Congo red from Egyptian textile factories instead of the traditional low-efficient treatment process with high efficiency, even at low concentrations, considerably decreasing environmental contamination. These environmentally acceptable and cost-effective adsorbents provide a long-term solution for treating textile effluent contaminated with Congo red. The chemical structures, performance during dye uptake, adsorption, and kinetics of the uptake process will be considered, including the mechanism of the process.

## Materials and experimental techniques

### Materials

Magnesium nitrate hexahydrate (Mg(NO_3_)_2_·6H_2_O) (purity 99.99%), aluminum nitrate nonahydrate (Al(NO_3_)_3_·9H_2_O) (purity 99.8%), nickel nitrate hexahydrate (Ni(NO_3_)_2_.6H_2_O) (purity 98.5%), anhydrous sodium hydroxide (NaOH) (purity 98%), sodium carbonate (Na_2_CO_3_) (purity 99.5%), and nitric acid (HNO_3_) (purity 70%) was obtained from Sigma Aldrich Chemicals Company (Egypt). Congo-red dye (CR) (Dye content ≥ 35%, Powder) obtained from Hisni for dyeing, finishing, and knitting, 10th of Ramadan-Sharqia, Egypt. Milli-Q water (19.1 mΩ·cm^−1^) was used.

### Mg/Ni/Al-LDH preparation

Two solutions were prepared to achieve the Ni-modified layered double hydroxide: solution A, and solution B^29,30^. Solution A comprising (Mg(NO_3_)_2_.6H_2_O, 38.3 g), (Ni(NO_3_)_2_.6H_2_O, 12.2 g), and (Al(NO_3_)_3_.9H_2_O, 23.9 g) dissolved in 0.15 L of deionized water. Solution B comprised sodium carbonate (Na_2_CO_3_, 10.6 g) and sodium hydroxide (NaOH, 4 g) dissolved in 0.15 L of deionized water. The solutions were mixed in a 0.5 L glass reactor containing 0.125 L of deionized while maintaining the alkalinity at 9–10. The obtained precipitate was robustly stirred overnight at 60 °C. Next, the product was filtered and washed thoroughly using hot deionized water to eliminate the extra and unreacted ions, and the water content was eliminated at 105 °C for one day. The achieved LDH was thermally treated (calcination) for 4 h at 300 °C to obtain the highest expected basic nature as reported^31^.

### Dye uptake experiments

The uptake of CR on the as-prepared Mg/Ni/Al-LDH and its thermally treated form was conducted by scattering an appropriate amount in 0.25 L of CR in 0.5 L flask and stirred at 150 rpm and 25 °C, followed by separation of the adsorbents by filtration.

The influence of the weights of the adsorbent was considered by utilizing 0.5–4 g of the two adsorbents in the presence of 200 ppm of Congo red dye in a 1000 mL solution. Similar conditions were used to determine the influence of pH of the medium at a pH range of 5–12. To evaluate the effect of time, the experimental adsorption test was conducted at 30, 60, 90, 120, 150, 180, 210, 240, 300, 330, and 360 min using 2 g/L of the prepared adsorbents in the attendance of 100 ppm of Congo-red dye as starting concentration. The influence of dye concentration was determined utilizing various dye concentrations (25, 50, 75, 100, 150, and 200 ppm) and 1 g/L adsorbents for 120 min. The residual amount of CR after each experiment was determined using a Camspec M501 UV/Vis spectrophotometer.

### Reusability studies

To test the recyclability of as-prepared Mg/Ni/Al-LDH and its thermally treated form, 0.5 g of the two compounds were mixed in 50 mL solution of Congo red dye (100 ppm) at pH of 7.6 and 7.9 respectively for 120 min at 25 °C. Then, the adsorbent was filtered and the amounts of the remaining dye were determined spectrometrically. The obtained adsorbents were collected and washed with the solution of nitric acid at pH 5, followed by washing with sodium hydroxide solution at pH of 8 in order to remove the adsorbed dye, and dried at 50 °C for 6 h^32^. Then, the experiment was repeated in five cycles, and the remaining concentration of the dye after each cycle was determined. After each experiment, the weight of the adsorbent was determined and an additional amount was added to compensate for the loss in weight due to the regeneration. The efficiency of removal was determined after each cycle for the two studied compounds.

## Results and discussion

### Characterization of the as-prepared and thermally treated Mg/Ni/Al-LDH

**Fig. 1:**
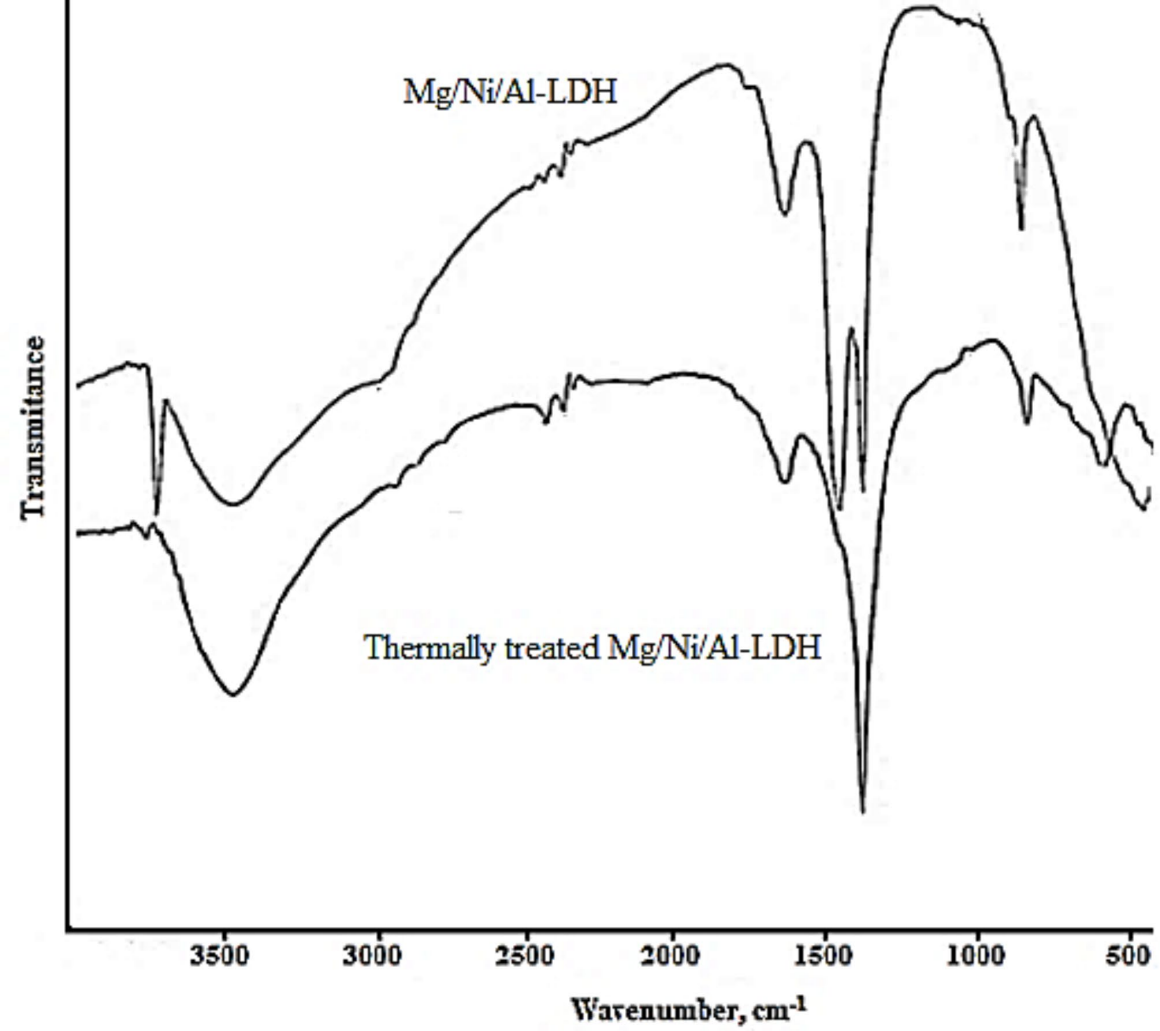
FT-IR spectra of Mg/Ni/Al-LDH before and after thermal treatment.

Figure 1 displays the FT-IR chart of the as-prepared and thermally treated Mg/Ni/Al-LDH at 4000 –500 cm^−1^ (Nicolet Magana-IR 750 spectrometer). The as-prepared Mg/Ni/Al-LDH displayed a sharp peak at 3650 cm^−1^ and a strong broad absorption peak centered at 3450 cm^−1^ which were ascribed for the different hydroxyl groups of the adsorbed water molecules and the surface (–OH) of the layered double hydroxide. The absorption peak at 1645 cm^−1^ was monitored for the deformation of water molecules^33^. The bands at lower frequencies of 1000 cm^−1^ were pointed for M-O and M-O-M vibrational modes in the LDH interlayers. Two absorption peaks appeared at 1385 cm^−1^ and 1375 cm^−1^ were pointed for the vibrational interaction of carbonate and nitrate anions, respectively. The FT-IR chart of the thermally treated Mg/Ni/Al-LDH represented a slightly narrow O-H stretching at 3450 cm^−1^. The band at 1385 cm^−1^ found in the as-prepared Mg/Ni/Al-LDH of carbonate anions disappeared and the band at 1375 cm^−1^ was shifted to the higher frequency of 1370 cm^−1^^34,35^ because of the disordered nature of the interlayer space. The vanishing of the absorption peak at 1385 cm^−1^ and the shifting of the band at 1375 cm^−1^ indicate that the thermally treated Mg/Ni/Al-LDH has a low abundance of nitrate anions in its interlayers with a complete decomposition of the carbonate anions.

**Fig. 2:**
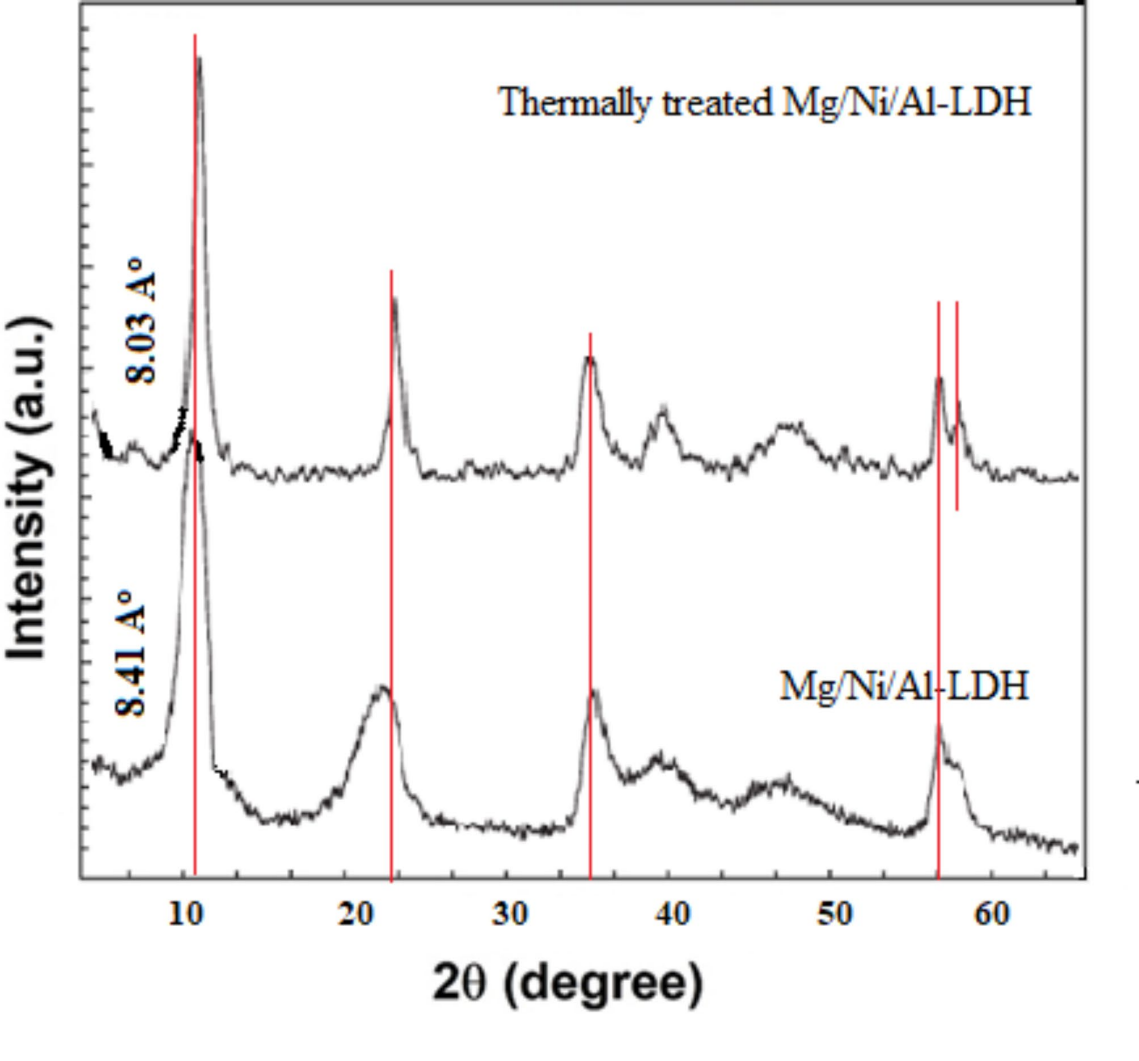
XRD patterns of Mg/Ni/Al-LDH before and after thermal treatment.

The powder XRD profile of the prepared Mg/Ni/Al-LDHs (Philips X’ Pert Pro Super X-ray diffractometer, Cu Kα source, λ = 1.5418 Å) was represented in Fig. 2. The XRD profiles of the Mg/Ni/Al-LDH and the thermally treated form showed three characteristic patterns at 10.51° (003), 23.2° (006), and 34.9° (009) which represented the layered form of the Mg/Ni/Al-LDH^36^ before and after the thermal treatment. After thermal treatment, besides the characteristic patterns at 11.07° (003), 23.2° (006), and 34.9° (009), a new pattern appeared at 57.7^o^ (110) which attributed to the formation of the corresponding metal oxide forms^37^. The peak at 2θ of 61° corresponded to (110)/(113) has appeared in both thermally treated and the as-prepared LDHs because it is a typical peak for LDHs. The appearance of the two crystalline planes of 003 and 006 in the thermally treated adsorbent can be ascribed to the temperature of the thermal treatment. The treatment was performed at 300 °C, which is lower than the reported temperature of the complete calcination of LDH. Hence, the obtained thermally treated HDH is expected to have a low abundance of nitrate and hydroxyl groups in the interlayers. Commonly, the XRD diffraction patterns of the as-prepared and the thermally treated Mg/Ni/Al-LDH form appeared in a narrow and sharp pattern indicating their well-crystallized arrangements^38^. The XRD patterns showed an increase in the crystallinity of the thermally treated Mg/Ni/Al-LDH which can be attributed to the formation of high crystalline mixed metal oxides (MMO)^39^ and appeared as an increase in the intensity of their XRD patterns (Fig. 2). The increase in the intensity of the thermally treated LDH may be attributed to the influence of long-term heating for 4 h at 300 °C, which improved the crystallinity of the formed MMO phase.

**Fig. 3:**
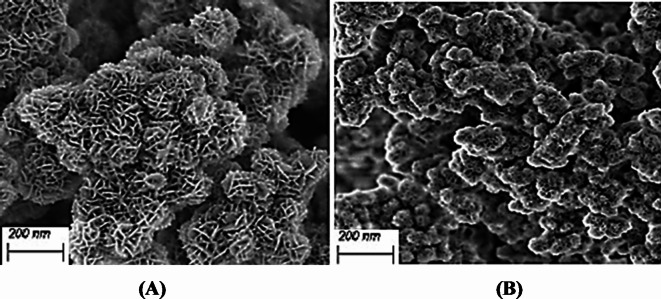
SEM profile of Mg/Ni/Al-LDH: (**A**) as-prepared Mg/Ni/Al-LDH, (**B**) thermally treated Mg/Ni/Al-LDH.

The SEM of the as-prepared Mg/Ni/Al-LDH was depicted in Fig. 3A using (JEOL JSM-JSM-6330 F analyzer). It is clear that the structure of the LDH has a flower-aggregated matrix with an irregular shape and obvious porous texture. It appeared clearly in the thermally treated Mg/Ni/Al-LDH the presence of two kinds of morphologies, sphere and plate-like structures (Fig. 3B). These morphologies facilitate the exposure of the adsorptive active sites and composite channels for the CR dye molecules in the medium. That remarkably improved the adsorption capacity of the adsorbent^40^. However, after the thermal treatment of the prepared LDH, the obtained particles were well-dispersed and the particle size was well-arranged as represented in Fig. 3B. The smaller size of the obtained particles and their porosity are expected to act as a good adsorbent for the pollutants in the wastewater. The crystallinity of the thermally treated LDH sample was much improved as represented in Fig. 3B which was in a well accordance with the corresponding XRD results in Fig. 2. It can be established that the thermal treatment results in a long-range order of the atoms in the compound structure, and thus increases the crystallinity^41^ as evidenced by the XRD patterns (Fig. 2). The high-temperature treatment breaks the layers of as-prepared Mg/Ni/Al-LDH into smaller aggregated particles, which was parallel to the reported results^39,42^.

**Fig. 4:**
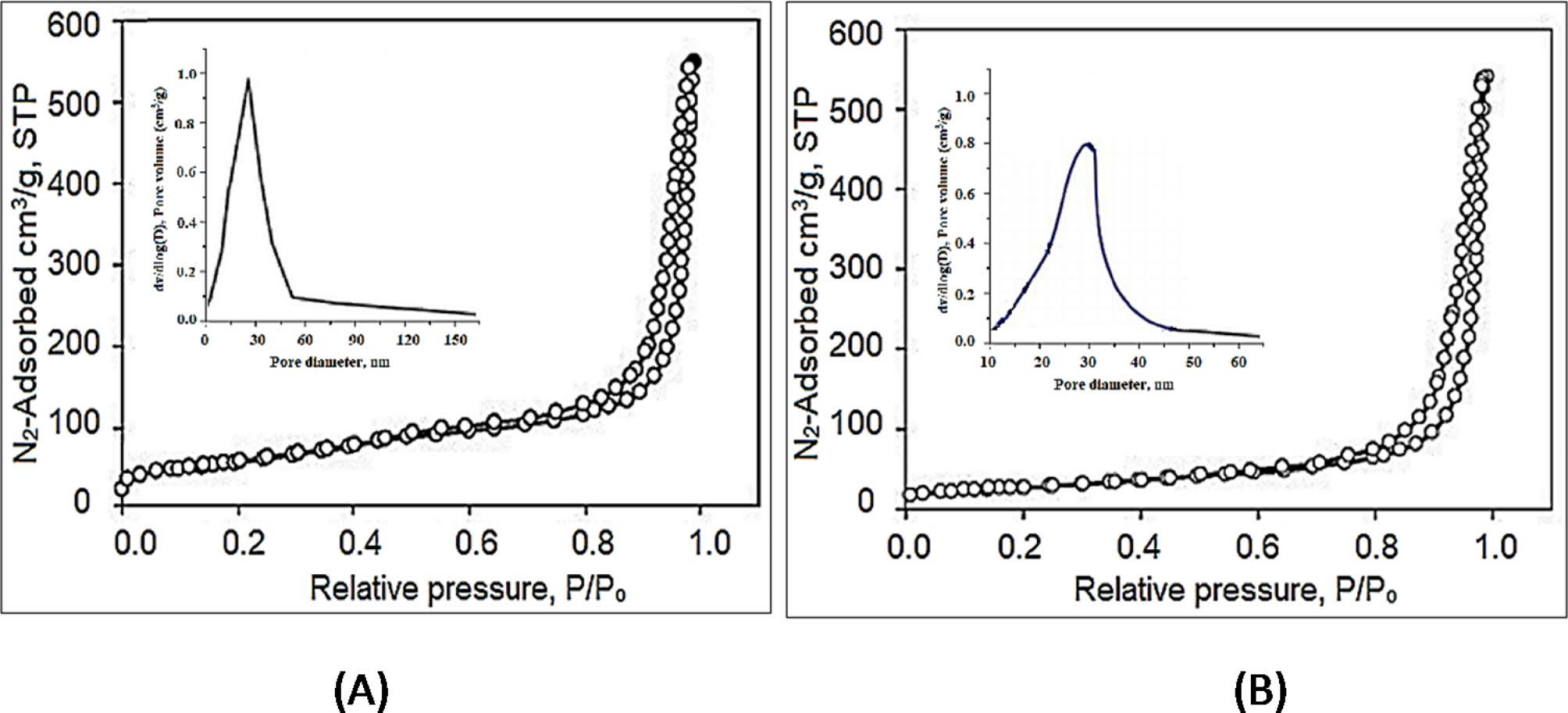
N_2_-adsorption/desorption profiles of (**A**) as-prepared Mg/Ni/Al-LDH, (**B**) thermally treated Mg/Ni/Al-LDH at 300 °C.

The BET analyses of the as-prepared and thermally treated compounds (Table 1) were measured (Fig. 4) using a Micromeritics 2020 HD88 analyzer, and the achieved parameters were registered in Table 1. The BET isotherms of the prepared compounds were of type IV accordingly IUPAC organization^1^ with H3 hysteresis loop. It was reported that Type IV hysteresis is associated with materials having a relatively large pore size distribution with a degree of surface heterogeneity. The difference in the heterogeneity of the surface can be ascribed to the different adsorption sites on their surface, which have different adsorption energies.

The surface pore area, pore volume, and average pore radius were determined from the data presented in Fig. 4 and reported in Table 1. The similarities of the adsorption/desorption characteristic profiles stem from similarities in the structure of the pores. This is explained by the mechanism of pore generation, as the produced pores are intra-particles, i.e., they are inside the compounds and so keep their adsorption properties regardless of the shape of their particle.

Figure 4 shows that the as-prepared compound has a significantly reduced surface area (109 m^2^/g) than the area of the thermally treated form (132 m^2^/g) at 300 °C. The onset graph of the average pore diameter of the two compounds showed a considerable increase in their values from 28.73 A° before calcination to 31.18 A° after calcination, with corresponding pore volume of 0.71 cm^3^/g to 0.85 cm^3^/g, respectively (Table 1). The calcination of the Mg/Ni/Al-LDH compound resulted in losing the surface and interfacial water molecules, Furthermore, the conversion of the metal hydroxides into oxide forms. That significant improvement in the surface area and pore volume of the resulting compound was described for the meso- and micropores formation^43^.

**Table 1:** Surface area and pore volumes of the prepared compounds.

Compounds	Surface area, m^2^/g	Average pore volume, cm^3^/g	Average pore diameter, (nm)
Mg/Ni/Al-LDH (as prepared)	109	0.71	28.73
Thermally treated Mg/Ni/Al-LDH	132	0.85	31.18

**Fig. 5:**
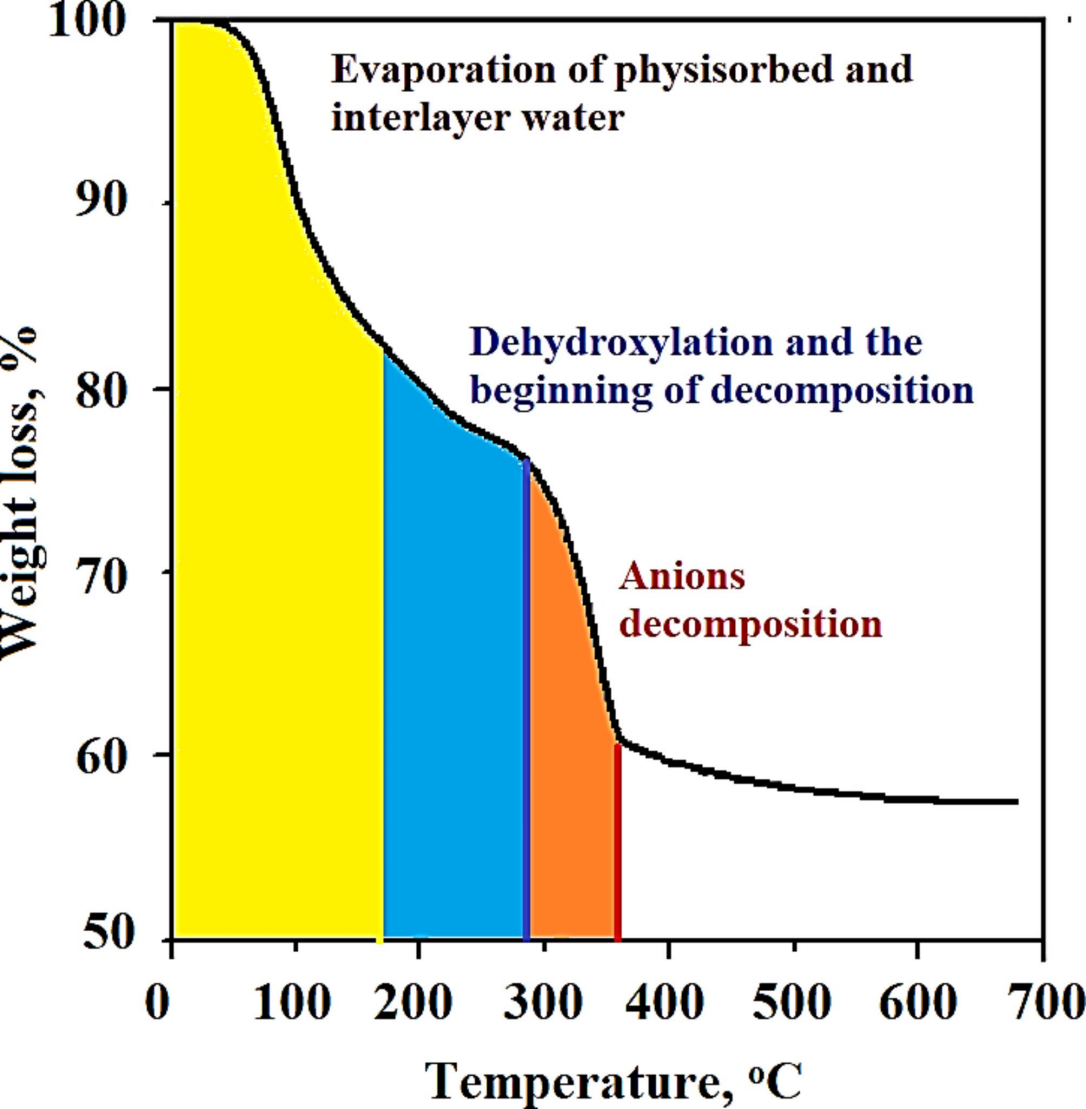
TGA curve of the as-prepared Mg/Ni/Al-LDH compound.

The thermal stability and decomposition of the as-prepared Mg/Ni/Al-LDH were examined by thermogravimetric analysis in the range of 25–700 °C, Fig. 5. As observed from Fig. 5, three regions can be identified representing the stages of the as-prepared Mg/Ni/Al-LDH decomposition. The first region is located between 25 and 200 °C and can be ascribed to the evaporation of H_2_O that is physically adsorbed water presented at the surface and in the interlayers^44^. The corresponding mass loss in this stage averaged 18% of the total sample weight. The second decomposition section is allocated at a temperature range of 200 °C to below 300 °C and is attributed to the de-hydroxylation of brucite-like layers (decomposition of the hydroxyl groups) and also indicates the starting of interlayer anions decomposition to form the oxide forms of the metal cations^45^. The process of anions decomposition is pointed beyond 300 °C which corresponds to the nitrate decompositions. The obtained results are comparable with reported data^46^.

### Properties of CR uptake on the as-prepared and thermally treated Mg/Ni/Al-LDH

**Fig. 6:**
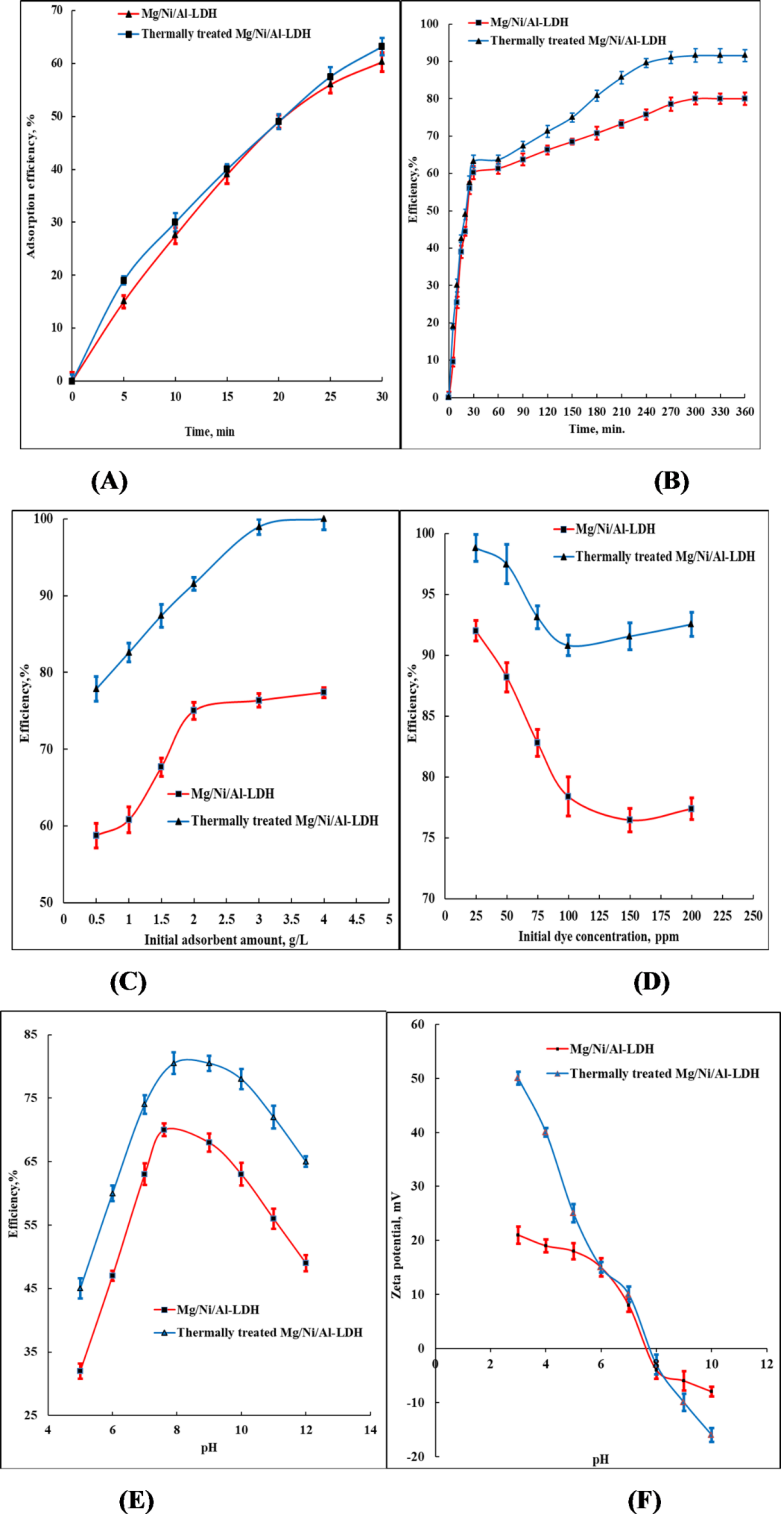
Effect of (**A**) immersion time at early adsorption stage (0–30 min), (**B**) immersion time at late adsorption stage (30–360 min.) (pH = 7, dye conc. 100 ppm, adsorbent weight 2 g/L, 25 °C), (**C**) initial adsorbent concentration (pH = 7, dye conc. 200 ppm, t = 270 min, 25 °C), (**D**) initial dye concentration (pH = 7, adsorbent weight 1 g/L, t = 120 min, 25 °C), and (**E**) pH (dye conc. 200 ppm, adsorbent weight 1 g/L, t = 270 min, 25 °C), on the uptake efficiency of CR using the as-prepared and thermally treated Mg/Ni/Al-LDH compounds from the aqueous medium, (**F**) point of zero charge of the as-prepared and thermally treated Mg/Ni/Al-LDH compounds.

Figure 6A, B illustrates the effect of time on the efficiency of the uptake process of CR using the as-prepared and thermally treated Mg/Ni/Al-LDH. Figure 6A represents the influence of immersion time of the as-prepared and thermally treated Mg/Ni/Al-LDH adsorbents on their adsorption efficiencies of the Congo red dye at 0–30 min. It’s noticeable that the adsorption process occurs quite quickly. The fast and rapid adsorption process can be attributed to two reasons; the first is the difference in the concentration gradients among the dye solution and the active adsorptive sites in the interlayers of the two adsorbents. The second is the comparatively rich and accessible active sites which strongly adsorb the dye molecules from the medium. The adsorption efficiencies of the as-prepared and thermally treated Mg/Ni/Al-LDH adsorbents reached 60.3% and 63.2%, respectively after 30 min^47^.

The gradual upsurge in the time of the uptake process from 30 min to 270 min increases the amounts of the removed dye, Fig. 6B. At 270 min, the efficacy of the process was increased to 75% in the case of the as-prepared Mg/Ni/Al-LDH, while it reached 90% using the thermally treated Mg/Ni/Al-LDH form. After 270 min exposure between the adsorbents and the dye molecules facilitates adsorption and desorption interactions leading to almost stable uptake of CR amount and consequently stable efficiencies^48^.

Figure 6C represents the influence of the as-prepared and thermally treated Mg/Ni/Al-LDH amounts through the uptake process of Congo-red dye molecules from the solution. The presence of small amounts of the adsorbents during the process had low uptake efficiency due to the lower number of accessible centers capable of uptake of the CR molecules, leading to fast saturation of the adsorbent surfaces^28,48^. The readily accessible uptake centers, as appeared in Fig. 6C, are higher in the case of thermally treated than the as-prepared Mg/Ni/Al-LDH because of the higher uptake efficiency of the thermally treated form during the process than that obtained by using the as-prepared Mg/Ni/Al-LDH. Increasing the amount of the used adsorbents during the process leads to stability in the obtained efficiency at higher values, indicating the increased number of the uptake centers by increasing the amount of the used adsorbents to 4 g/L.

The effect of the initial pollutant conc. on the uptake efficiency of the prepared compounds is represented in Fig. 6D. At low initial concentrations, the efficiencies of the uptake process reached 95% and 100% using the as-prepared and thermally treated Mg/Ni/Al-LDH form, respectively. While the gradual increase in the initial dye conc. has a descending effect on the obtained uptake efficiencies. That can be ascribed to the fast uptake of the low-concentration dye molecules by the comparatively large number of uptake centers on both compounds. Increasing the dye concentration leads to saturation of the uptake centers on the surfaces of the adsorbent and appears as a straight part in Fig. 6D at an initial dye concentration of 150 ppm. The thermally treated Mg/Ni/Al-LDH form is highly effective during the uptake process of CR than the as-prepared Mg/Ni/Al-LDH. That can be revealed by the higher number of accessible uptake centers on the thermally treated form as the influence of the thermal treatment.

Generally, the alkalinity and acidity of the medium have a high effect on the uptake efficacy of the LDH compounds. The LDHs have alkaline characters because of the attendance of the hydroxyl groups on their surfaces, and also because of the occurrence of the metal oxide forms increase of the thermally treated forms. Herein, the measurements were performed at pH starting from 5 to 12, due to the acidic medium dissolving the LDHs. Figure 6E represents the effect of the alkalinity of the medium on the uptake efficacy of the as-prepared, and thermally treated Mg/Ni/Al-LDH form. At a pH of 5, the uptake efficiencies were at the lowest values due to blocking the most accessible uptake centers on the adsorbent surfaces resulting from the neutralization with H^+^ ions^49^.

Increasing the pH of the medium around 7–8 decreases the concentration of H^+^ ions, which consequently increases the number of accessible centers and the uptake efficiencies of the studied adsorbents. That can be referred to as the point of zero charge of the two compounds. At the alkaline medium (pH 8–12), the adsorption efficiencies were decreased by increasing the alkalinity of the medium because the adsorptive active sites at the adsorbent surface acquire negative charges and the sulfonate groups of the dye molecules ionized to have negative charges. The electrostatic repulsion among the similar negative charges of the adsorbents and the adsorbates decreases the adsorption efficiencies considerably^50^.

The point of zero charges (PZC) of the as-prepared and the thermally treated form of the prepared LDH was obtained from the measurements of the potential of the compounds in the aqueous media at a wide range of pH, Fig. 6F. The potentiometric method was applied by titration of the prepared LDH compounds in suspension using HCl solution (1 M) or NaOH solution (1 M) while measuring the potential (mV) and the pH of the solutions. PZC of the two adsorbents was determined as the pH value corresponding to the potential of the LDH suspensions is zero (mV)^51^. It is clear that the points of zero charges of the two compounds are located at the alkaline region which revealed that the compounds are charged by positive charges at pH values lower than 7, and had zero charges at 7.6 for the as-prepared LDH, while the thermally treated form of LDH had zero charge at 7.9. The increase in the Pzc and consequently the positive surface charges for the thermally treated LDH can be ascribed to the loss of counter-nitrate ions after the calcination process. The uptake efficiencies were allocated at 84% and 92.5% using the as-prepared and thermally treated Mg/Ni/Al-LDH forms, respectively.

It is observable from Fig. 6A–E that the thermally treated Mg/Ni/Al-LDH form has higher efficiency during the uptake process of CR than the as-prepared Mg/Ni/Al-LDH form. It was reported that the thermal treatment of the hydrotalcite compound resulted in the production of various types of pores in their crystalline framework^52^. The calcination of Mg/Ni/Al-LDH leads to dehydration of surface and interfacial water molecules, and the creation of various kinds of metal oxides within its structure. The oxide forms performed an effective role in the uptake process due to the creation of readily accessible centers capable of attaching to CR molecules.

### Adsorption study

**Fig. 7:**
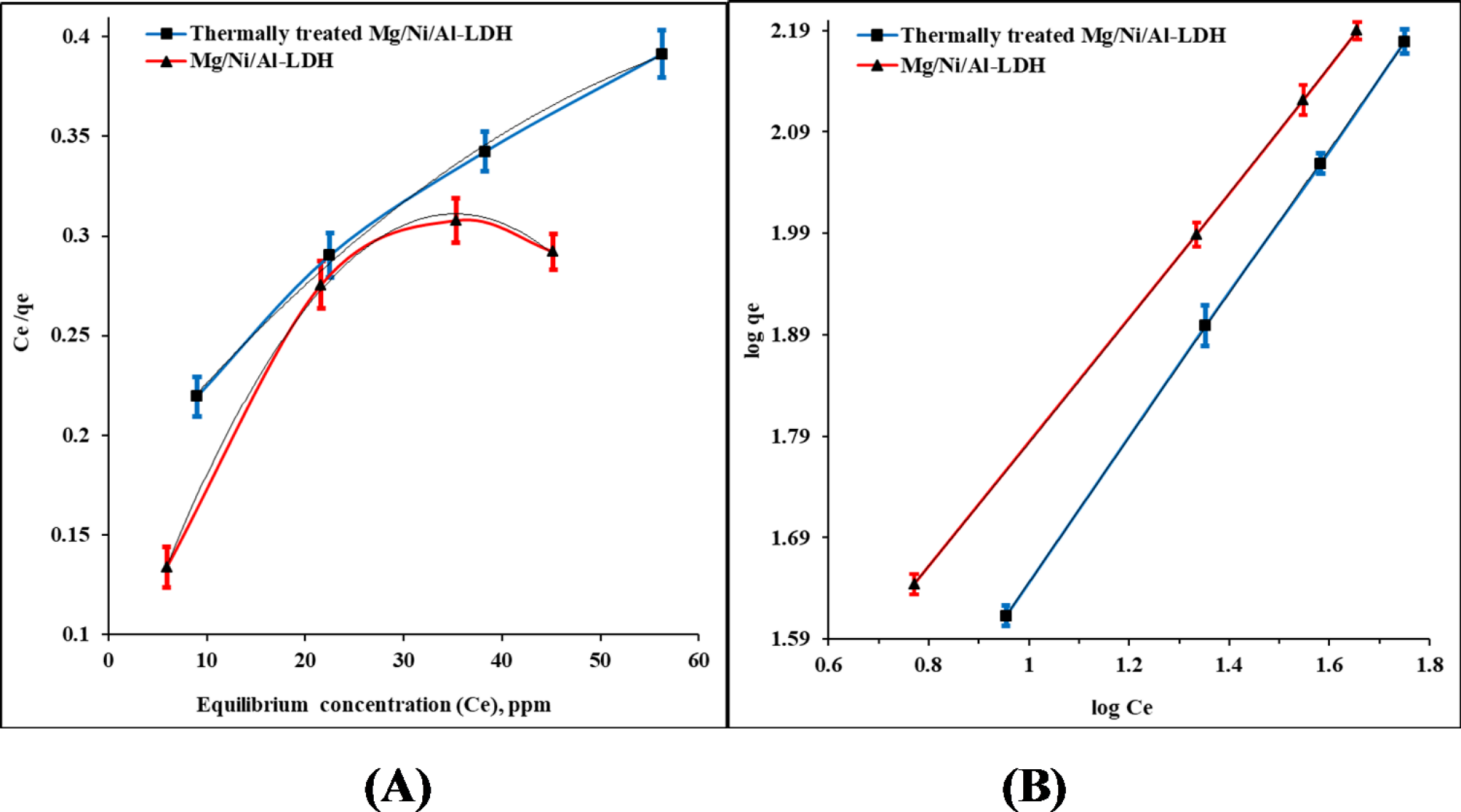
(**A**) Langmuir, (**B**) Freundlich, adsorption isotherms representation of CR uptake on the prepared compounds.

The uptake mechanism of CR molecules on the as-prepared and thermally treated Mg/Ni/Al-LDH forms was investigated by evaluating equations of the state of two adsorption isotherm models, Langmuir and Freundlich adsorption isotherm models, Table 2. Figure 7A represents the variation of the variable (*C*_*e*_*/q*_*e*_) by the values of the equilibrium concentration (*C*_*e*_) of CR at equilibrium during the uptake process. The graphical relation at process time between 30 and showed deviation from the linear trend with correlation coefficients (R^2^) at 0.753, and 0.9771 for the as-prepared and thermally treated Mg/Ni/Al-LDH forms, respectively, showing the unsuitability of this model for describing the process^53^ Investigating the experimental results by applying the equation of state for the Freundlich model^54^. Figure 7B gave unity correlation coefficients of 1, Table 2. Consequently, the mechanism of CR uptake using the as-prepared and thermally treated Mg/Ni/Al-LDH compounds follows the postulates of the model step. The model also suggests the presence of electrostatic interactions among the attached CR dye ions on the surface of the adsorbents^55^. The electrostatic interactions occur between the –OH moieties on the LDHs surfaces and the different functional groups of the dye molecules. The higher values of n than unity predict the strong attachment of dye molecules on the prepared adsorbent surfaces^56^. Hence, the Freundlich adsorption model parameters revealed that the uptake of the CR molecules is tightly attached to the surface of the as-prepared and thermally treated Mg/Ni/Al-LDH forms in the attendance of different interactions between the attached molecules due to the difference in the chemical structures of the hosting centers on the surface of the two adsorbents^57^.

### Kinetic study

The kinetic study of CR adsorption using the as-prepared and thermally treated Mg/Ni/Al-LDH adsorbents was divided into two parts, the first is at the early stage of adsorption which occurred at the first 30 min of the process, and the second is the adsorption at equilibrium which occurred from 30 min until equilibrium.

**Fig. 8:**
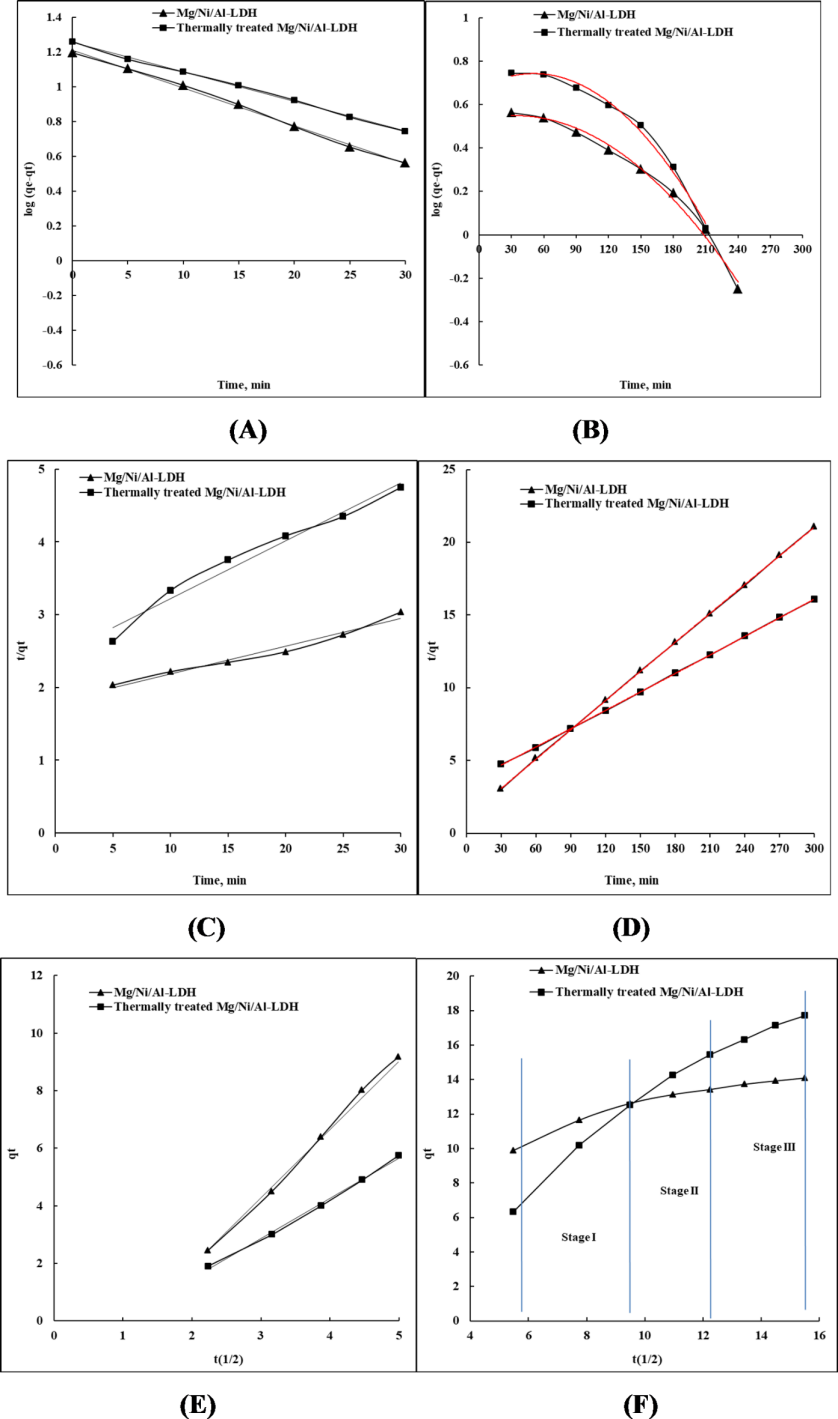
(**A**) Pseudo-first-order for the early-stage adsorption from 0–30 min, (**B**) Pseudo-first-order for the late stages adsorption from 30–300 min, (**C**) pseudo-second-order for the early-stage adsorption from 0–30 min, (**D**) pseudo-second-order for the late stages adsorption from 30–300 min, (**E**) Inter-particle diffusion models for the early-stage adsorption from 0–30 min, (**F**) Inter-particle diffusion models for the late stages adsorption from 30–300 min of CR on the as-prepared and thermally treated Mg/Ni/Al-LDH forms.

Figure 8A represents the different kinetic evaluations of the uptake process of CR molecules on the as-prepared and thermally treated Mg/Ni/Al-LDH adsorbents for the early-stage adsorption at 0–30 min, while Fig. 8B profiled the evaluation of the experimental data at 30–240 min. Analysis of Fig. 8A showed that the process of adsorption at the early stage duration has correlation coefficients (R^2^) values of 0.9974 and 0.9984 for as-prepared and thermally treated Mg/Ni/Al-LDH adsorbents, respectively, while Fig. 8B resulted in R^2^ values at 0.9124 and 0.8702. Comparing the results of the obtained R^2^ values indicates that the process of adsorption obeys the pseudo-first-order isotherm during the early adsorption stages while increasing the processing time deviates the process from the pseudo-first-order representation (Table 2). The agreement of the adsorption process at the early stage is due to the fast adsorption of the dye molecules due to the high accessibility of the adsorption sites at the surface of the two adsorbents.

For describing the kinetic isotherm of the adsorption process, a pseudo-second-order isotherm was applied. The early-stage adsorption process showed deviation from the isotherm as their R^2^ values were 0.9721 and 0.9722 for as-prepared and thermally treated Mg/Ni/Al-LDH, respectively (Fig. 8C). At the late stages (30–300 min) (Fig. 8D), the correlation coefficients of as-prepared and thermally treated Mg/Ni/Al-LDH adsorbents represented the agreement between the experimental and the modeled data as their R^2^ values were about 1.

Accordingly, the equilibrium concentrations of CR were pointed from the model profile at 15.04 and 19.15 (mg/g) for the studied adsorbents, which were comparable to the experimental values (Table 2). The obtained rate constant (K_2_) from the model revealed the faster rate process of the dye molecules using thermally treated Mg/Ni/Al-LDH compound than that happens by using the as-prepared Mg/Ni/Al-LDH form (Table 2).

It was reported that the uptake process occurred through several stages according to the chemical structure of the adsorbent substrate and the adsorbed molecules^58,59^. The stages of the uptake process of CR molecules on the as-prepared and thermally treated Mg/Ni/Al-LDH compounds were determined using the inter-particle diffusion model^60,61^. The evaluation of the model using the measured uptake data resulted in different parameters listed in Table 2.

The steps of the uptake process can be elucidated using the inter-particle diffusion adsorption model (Fig. 8E, F), which describes the effective stages that influence the rate of the uptake process of CR molecules on both of the as-prepared and thermally treated Mg/Ni/Al-LDH forms^62^. The pre-stage of adsorption during 0–30 min showed a simple arrangement of CR molecules at the adsorbent surface, Fig. 8E.

The long-term uptake process proceeded through three stages, Fig. 8F. The first stage is a simple attachment of the dye molecules to the uptake sites on the two adsorbent surfaces^63^. The rate constants of the first stage using the two adsorbents were showed a faster manner in the case of thermally treated Mg/Ni/Al-LDH form (k_int1_ = 1.5519 mg/g.min) than the as-prepared form (k_int1_ = 0.6857 mg/g.min), Table 2. That can be attributed to the larger surface area and pore volume of the thermally treated Mg/Ni/Al-LDH form^64^. The second period represented the diffusion of the CR molecules into the layers of the two compounds^63^, which was characterized by a slow rate of diffusion in the case of the as-prepared LDH (k_int2_ = 0.2023 mg/g.min) compared to the comparatively faster process in the case of the thermally treated LDH (k_int2_ = 0.7068 mg/g.min), Table 2. The third stage in this model is the precipitation of the residual dye molecules in the adsorbent pores to form multilayers of the dye molecules onto the adsorbent with comparatively higher rate constants^64^. The precipitation rate constant (K_int3_) of dye molecules in the case of the as-prepared LDH was 0.9847 mg/g. min, while K_int3_ in the case of the thermally treated LDH was 1.0673 mg/g.min, Table 2. Comparing the rate constants (K_int_) of the three stages of the inter-particle diffusion model describing the adsorption process of CR dye onto the prepared adsorbents in Table 2 revealed that the second stage of the inter-particle diffusion model, the diffusion step, is the rate-determining stage of the uptake process own to its low K_int_ values for both adsorbents. Table 2 lists the obtained parameters after applying the equation of state of the model.

**Table 2:** Adsorption and kinetic parameters of the uptake process of CR on the prepared compounds.

Model	As-prepared Mg/Ni/Al-LDH	Thermally treated Mg/Ni/Al-LDH	Equation of state
q_e (Exp.)_ (mg/g)	15.71	18.19	
Models of adsorption isotherms			
Langmuir	R^2^ = 0.9989	R^2^ = 0.9982	$\frac{{{C_e}}}{{{q_e}}}=\frac{1}{{{q_{\hbox{max} }}{k_l}}}+\frac{{{C_e}}}{{{q_{\hbox{max} }}}}$
Freundlich	R^2^ = 1.0 *n* = 1.41 k_f_ = 13.51 ppm	R^2^ = 1.0 *n* = 1.63 k_f_ = 17.67 ppm	$\ln {q_e}=\frac{1}{n}\ln {C_e}+\ln {k_f}$
Models of kinetic isotherms			
Pseudo first order	R^2^ = 0.9919	R^2^ = 0.9928	$$\begin{gathered} \log \left( {q_{e} - q_{t} } \right) = \log q_{e} \hfill \\ \quad \quad \quad \quad \quad \quad - \frac{{k_{1} }}{{2.303}}t \hfill \\ \end{gathered}$$
Pseudo second order	k_2_ = 3.91 × 10^− 3^ (g/mg min) q_e_ = 15.04 (mg/g) R^2^ = 1.0	k_2_ = 8.02 × 10^− 4^ (g/mg min) q_e_ = 19.15 (mg/g) R^2^ = 0.9999	$\:\frac{t}{{q}_{t}}=\frac{1}{{k}_{2}{q}_{e}^{2}}+\frac{1}{{q}_{e}}t$
Inter-particle diffusion	*Stage 1 (adsorption)* k_int1_ = 0.6857 (mg/g.min) R^2^ = 0.9968	*Stage 1 (adsorption)* k_int1_ = 1.5519 (mg/g.min) R^2^ = 0.9975	$\:{q}_{t}={k}_{int}{t}^{\frac{1}{2}}+C$
	*Stage 2 (diffusion)* k_int2_ = 0.2023 (mg/g.min) R^2^ = 0.9988	*Stage 2 (diffusion)* k_int2_ = 0.7068 (mg/g.min) R^2^ = 0.9999	
	*Stage 3 (precipitation)* k_int3_ = 0.9847 (mg/g.min) R^2^ = 0.9893	*Stage 3 (precipitation)* k_int3_ = 1.0673 (mg/g.min) R^2^ = 0.9965	

*R*: correlation coefficient, *n*: reactive site intensity, *k*_*f*_: Freundlich adsorption coefficient constants that belonged to the adsorption capacity (mg/g), k_1_: pseudo-first-order rate constant (min^−1^) *k*_*2*_: pseudo-second-order rate constant (g/mg.min), *q*_*e*_: adsorption capacity at equilibrium (mg/g), *q*_*t*_ adsorption capacity at time t (mg/g), *q*_*max*_ maximum adsorption capacity (mg/g), *C*_*e*_: dye concentration at equilibrium (mg/L), *k*_*int1*_: rate constant of adsorption (mg/g.min), *k*_*int2*_: rate constant of diffusion (mg/g.min), *k*_*int3*_: rate constant of precipitation steps (mg/g.min).

### Reusability study

**Fig. 9:**
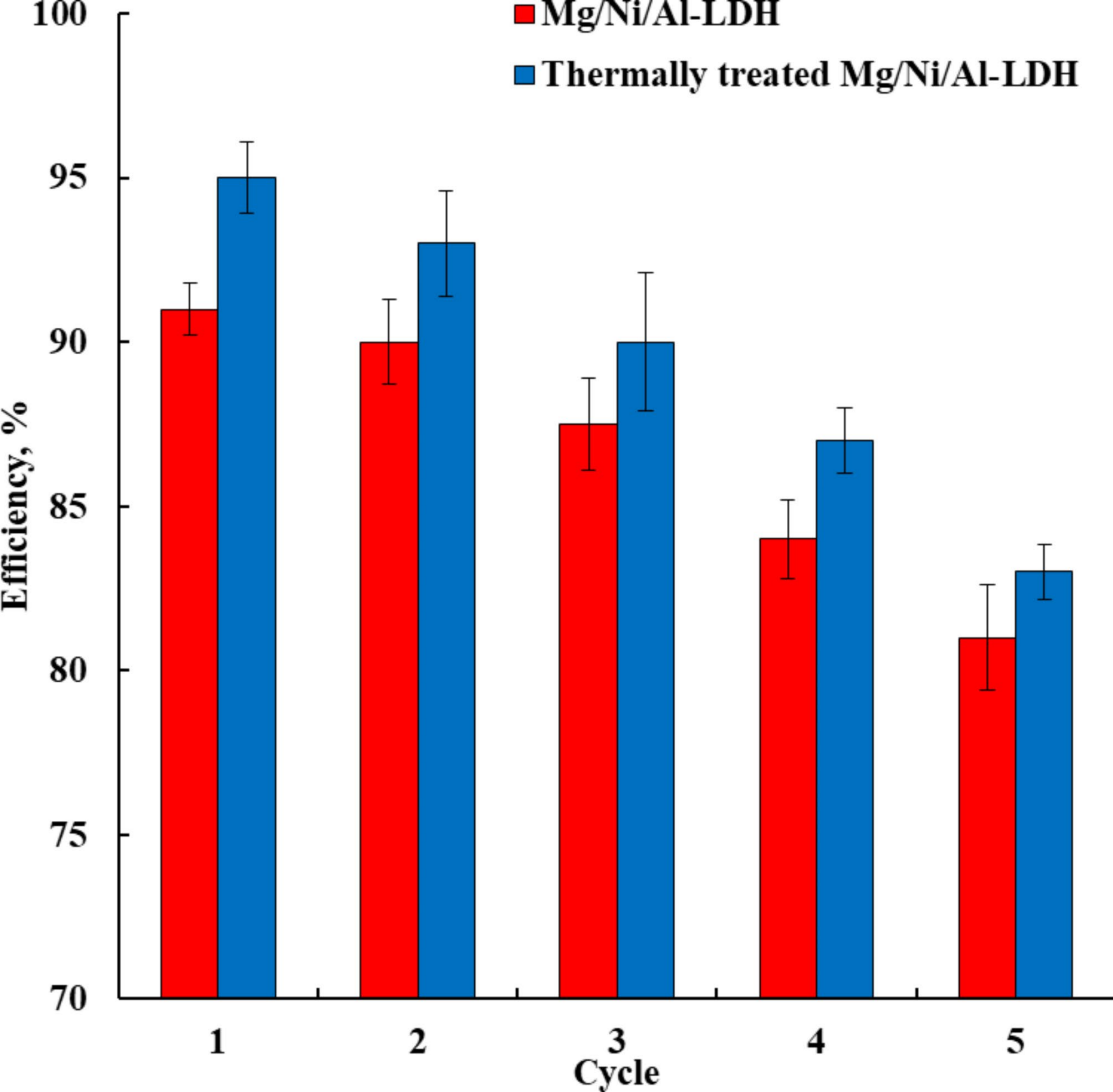
The recyclability of the as-prepared and thermally treated Mg/Ni/Al-LDH compounds during adsorption of Congo red dye at the optimum conditions.

The ability to reuse the adsorbents after the adsorption of pollutants reflects the economic impact of the process and the adsorbents. Herein, the regeneration of the prepared adsorbents was performed in two consecutive stages. The first was acidic washing to remove the adsorbed dye molecules from the adsorbent surfaces. That leads to the deactivation of the active sites on their surface due to their interaction with the nitric acid. The second stage was washing with NaOH solution to remove the attached nitric acid from the surface of the adsorbents to activate their active sites. Hence, the two-stage regeneration was important in their operation and order of application. The reusability of the prepared adsorbents is represented in Fig. 9. It is clear that the first cycle of the as-prepared and thermally treated Mg/Ni/Al-LDH forms comprised adsorption efficiencies at 91% and 95% respectively, which were slightly decreased by increasing the number of adsorption cycles up to 5 cycles to reach 81% and 83%, respectively. The results showed the good reusability of the prepared adsorbents during the treatment of wastewater polluted by Congo red dye from industrial applications.

### Real water samples treatment

Three water environments, viz. drinking, sea, and industrial wastewater from Hisni for dyeing, finishing, and knitting factory as real samples were designated to examine the possible application of thermally treated Mg/Ni/Al-LDH form as an active adsorbent for CR uptake by utilized the batch removal experiments. The 50–150 ppm CR spiked water samples were treated with 0.5 g of thermally treated Mg/Ni/Al-LDH form using optimal regulating experimental conditions (pH 2 and 35 min). The results of this examination are recorded in Table 3 and obvious that, the removal efficacy (%) values of CR by thermally treated Mg/Ni/Al-LDH form were recognized to reach the ranges 98.3–90.3% (50 mg/L CR dye), 88.6–85.3% (100 mg/L CR dye) and 95-89.7% (150 mg/L CR dye) from drinking, sea and wastewaters, correspondingly. It could be established from Table 3 that the greatest CR dye uptake amounts were associated with 50 ppm. The described data from this evaluation confirm that the thermally treated Mg/Ni/Al-LDH form an effective, economical, recyclable, and designed multi-functionalized structural adsorbent for environmental water remediation from CR dye, a persistent and toxic dye pollutant.

**Table 3:** Efficiency of thermally treated Mg/Ni/Al-LDH adsorbent during the removal of Congo red dye from three real water samples (processing conditions: water volume = 25 mL for 120 min in the presence of 50–150 ppm CR dye concentration using 0.5 g of thermally treated Mg/Ni/Al-LDH).

Conc. of Congo red, ppm	Removal efficiency, %		
	Tap water	Sea water	Wastewater
50	98	88.6	95
100	92.1	87.9	92.1
150	90.3	85.3	89.7

### Mechanism of uptake process

Congo-red dye is an anionic azo-dye compound comprising naphthalene moieties and biphenyl junctions in addition to the amino group and sulfate group in the form of sodium salt. The crystalline lattice of the as-prepared LDH comprises planer sheets of Mg and Al linked by oxygen atoms and covered by a hydroxyl groups-rich layer on the surface countered by nitrate ions. While the thermally treated form of the prepared LDH has successive layers of Al and Mg oxides without the presence of water molecules.

The uptake efficiencies of as-prepared and thermally treated forms of the prepared LDH are presented in Fig. 6. It is clear that the uptake efficiency of the as-prepared form of LDH was increased considerably upon thermal treatment at 300 °C. The mechanism of the Congo red adsorption on the as-prepared and thermally treated LDH will be discussed based on the FT-IR, and XRD measurements.

The adsorption efficiency versus pH relationship of Congo red dye onto the as-prepared and thermally treated form of the prepared LDH illustrated in Fig. 6E represented that the adsorption was increased gradually by increasing the pH of the medium to reach the maximum at pH values of 7.6 and 7.9 for as-prepared and thermally treated LDH forms, which is corresponded to their pHs of Pzc. Further increase in the pH declined the efficiency values. That illustrates the electrostatic interaction as an influential mechanism that occurred during the adsorption of Congo red dye molecules onto the two LDH compounds via the attraction of the Congo red molecules by the metal cations of Mg^2+^ and Al^3+^ presented on the surface of the adsorbents.

**Fig. 10:**
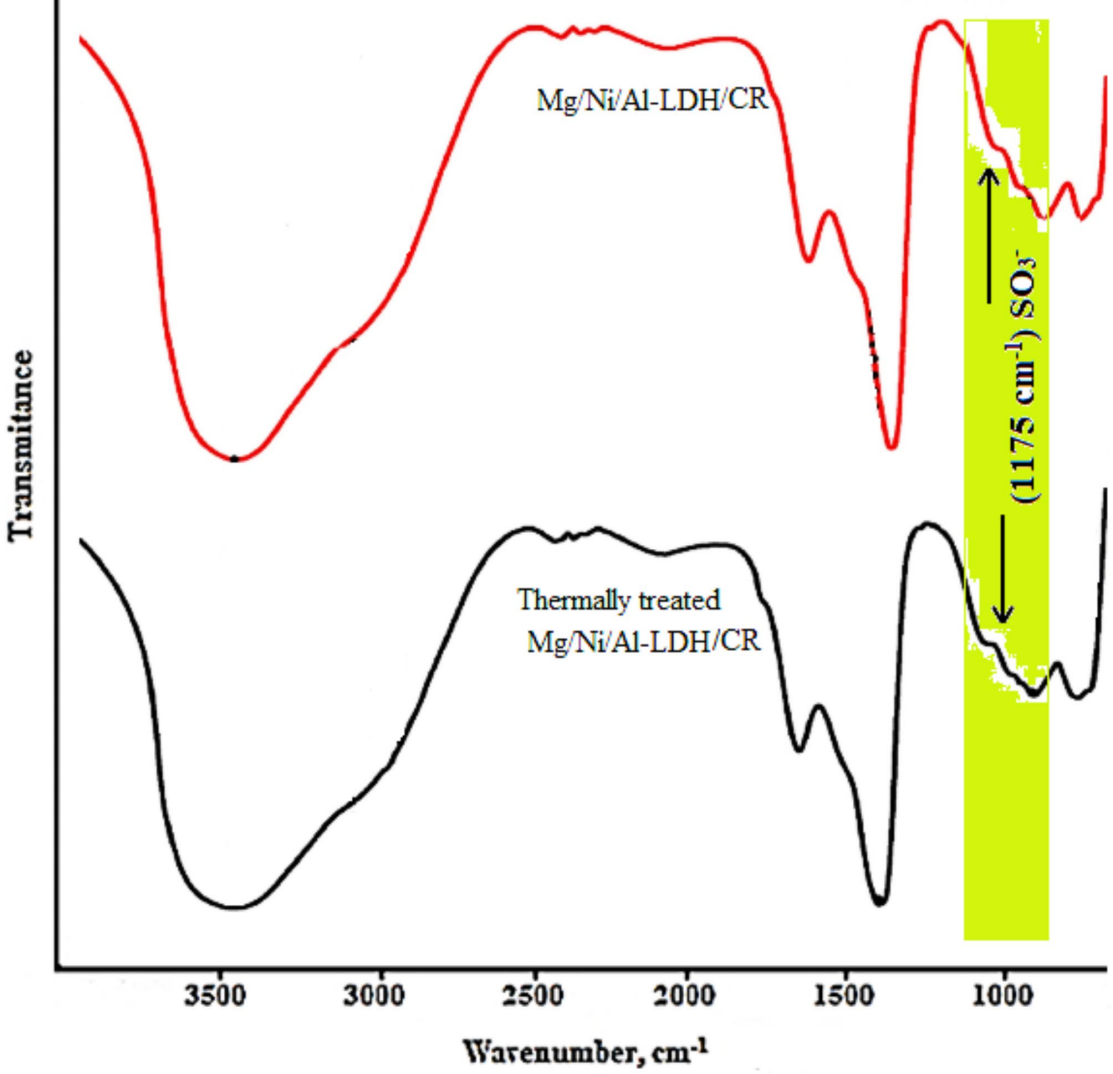
FT-IR spectra section of the Congo red dye loaded on the prepared compounds.

The FT-IR spectra of the as-prepared and thermally treated LDH adsorbents after adsorption of the Congo red from the medium were represented in Fig. 10. The spectra showed the appearance of two new characteristic absorption bands at 1047 cm^−1^ and 1175 cm^−1^, which defined the stretching vibrations of the SO_3_^−^ group of the Congo red dye molecules^67^. The presence of the two bands exposed the successful capturing of dye molecules by the two forms of the LDH. The attachment of the dye molecules is accompanied by the replacement of nitrate (NO_3_^−^) groups in the LDH layers by the sulfate (SO_3_^−^) groups of the CR molecules according to ion exchange mechanism^68^.

**Fig. 11:**
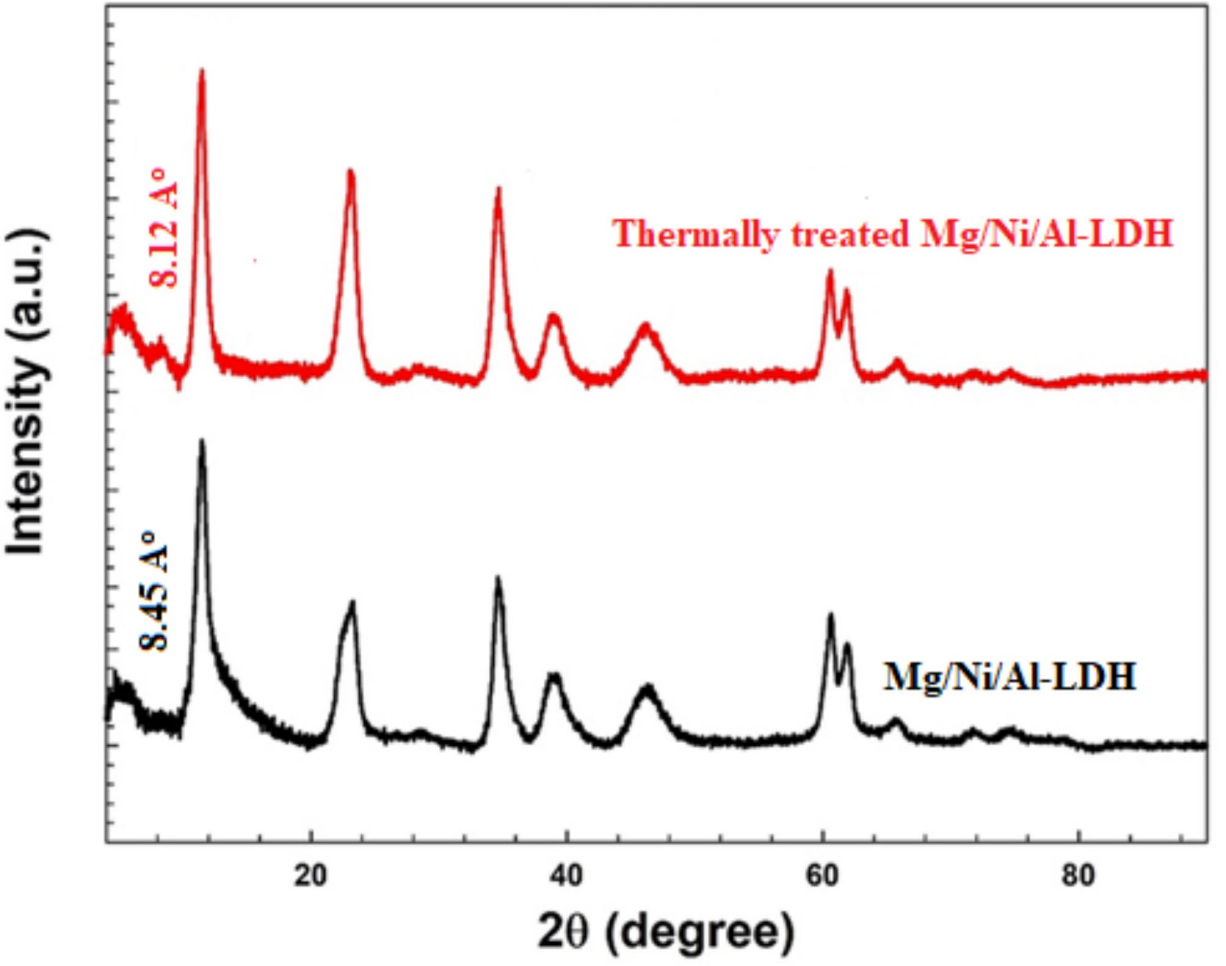
XRD patterns of the prepared compounds after adsorption of Congo red dye.

The XRD patterns of the prepared adsorbents before the adsorption of CR dye represented *d*-spacing values at 8.41 Å (2θ = 10.56°) and 8.03 Å (2θ = 11.07°) for the as-prepared and thermally treated compounds (Fig. 2), respectively. After the adsorption of CR dye molecules, the XRD patterns of the LDH compounds under investigation showed a slight increase in the *d*-spacing values to reach 8.45 Å (2θ = 10.51°) and 8.12 Å (2θ = 10.95°) for the as-prepared and the thermally treated LDH compounds. It was reported that the position of the adsorption of the dye molecules determines the manner of deviation of the d-spacing of the different LDH compounds. In the case of the occurrence of the adsorption at the surface of the LDH adsorbent, the d-spacing of the clay shifted towards lower values. That was ascribed to the squeezing that occurred at the surface of the crystallite LDH adsorbents leading to the decrease in their d-spacing values^69^. On the other hand, the occurrence of the adsorption of the dye molecules between the interlayers of the LDH adsorbents resulted in increasing the d-spacing values. The increase in the d-spacing is due to expanding the distances between the crystallite layers of the LDH adsorbents as the dye molecules entered within these layers^70^. The finding herein showed an increase in the d-spacing of the as-prepared LDH from 8.41 Å to 8.45 Å, and in the case of the thermally treated LDH, the d-spacing increased from 8.03Å to 8.12Å. That reached the conclusion of the adsorption of CR dye molecules on the prepared LDH adsorbents intercalated between the interlayer of the adsorbents layers. Comparing the XRD patterns in Figs. 2, and 11, the as-prepared and thermally treated LDH adsorbents after adsorption of CR dye molecules, the intensities pointed for several diffraction peaks were decreased. This can be originated to the adsorbed CR molecules in the interlayers which results in decreasing the diffraction pattern intensities resulting from certain crystalline planes^39^.

The results of the analysis for the different LDHs loaded by the Congo red dye revealed that the mechanism of the adsorption is composed of two main mechanisms. Firstly, the electrostatic interaction between the metal cations (Mg^2+^, Al^3+^) and the negatively charged sulfate groups of the dye molecules. Second, the ion exchange between the counter ions groups (NO_3_^−^) on the surface and in the interlayer spaces between the LDH layers and the sulfate groups of the dye molecules. According to the kinetic study, the adsorption of the dye molecules followed the Freundlich adsorption model which suggests the presence of electrostatic interaction on the various accessible uptake centers with different strengths based on the charge and availability of the different centers^71,72^. It was reported that the presence of Ni- cations in the prepared adsorbents has a minor influence on their adsorption efficiency, but its role is to facilitate the adsorption through enhancement of the electrochemical properties of the adsorbent which improves its adsorption ability during the different adsorption mechanisms^73^. Furthermore, hydrogen bonds take place during the interaction between the two moieties through the formation of the bonds between the sulfate groups and the surface hydroxyl groups. Also, two types of interactions occur; the first is the metal-nitrogen bonding between the nitrogen atoms of the dye molecules and the metal cations presented in the framework of the adsorbents The second is the Yushida hydrogen bonding between the hydrogen of the surface hydroxyl groups and the electron clouds presented in the phenyl groups of the dye molecules^74^.

**Fig. 12:**
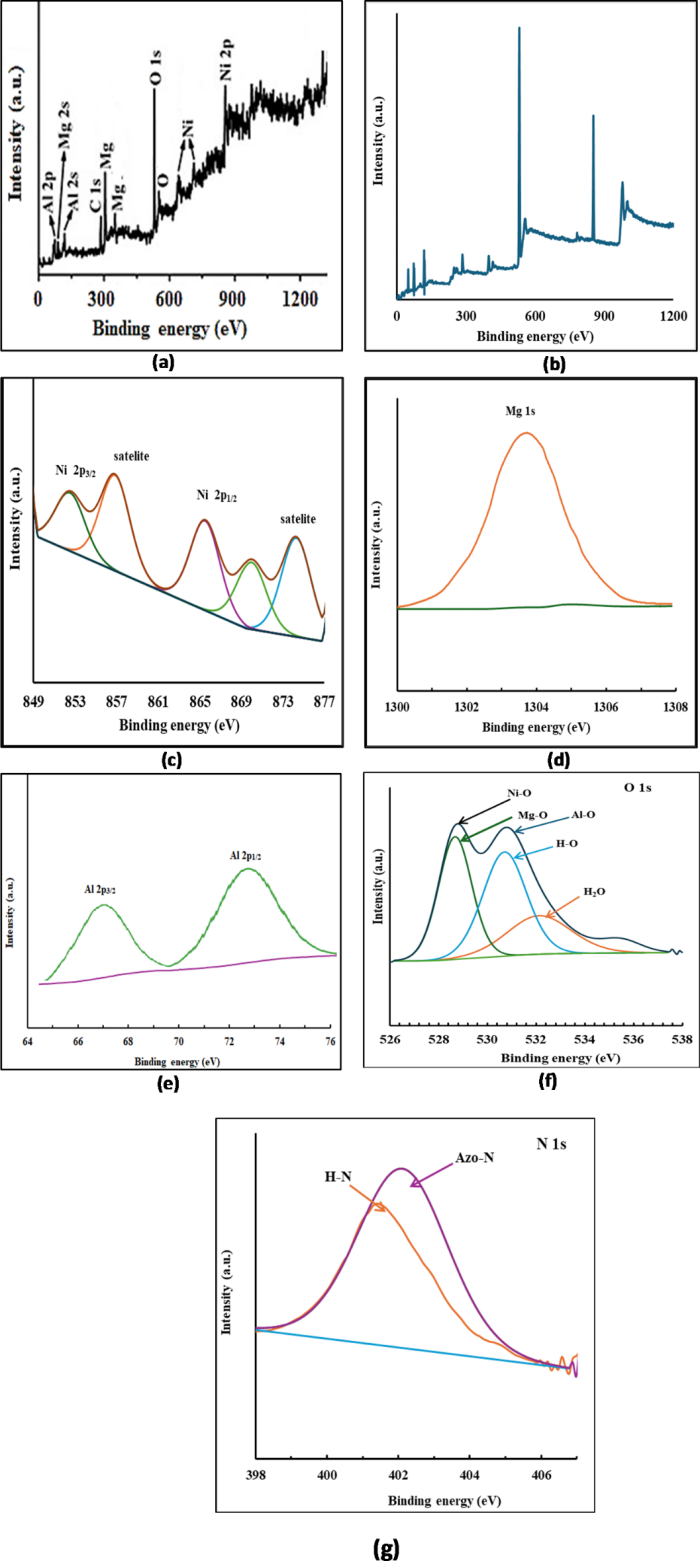
XPS survey spectrum of thermally treated Mg/Ni/Al-LDH (**a**), survey spectrum of thermally treated Mg/Ni/Al-LDH after CR adsorption (**b**), high-resolution XPS spectra of Ni 2p (**c**), Mg 1s (**d**), Al 2p (**e**), O 1s (**f**), and N 1s (**g**) of thermally treated Mg/Ni/Al-LDH after CR adsorption.

The presence of the different elements and their states on the surface of thermally treated Mg/Ni/Al-LDH is represented in the full spectrum survey of X-ray photoelectron spectroscopy (XPS), Fig. 12a. The presence of Ni, Mg, Al, and O elements represented the spectrum. After adsorption of Congo red dye on the thermally treated Mg/Ni/Al-LDH (Fig. 12b), the full spectrum survey displayed the presence of Ni, Mg, Al, and O atoms with slight shifting in their energy binding compared to the unloaded LDH, in addition to the appearance of C, and N elements. It was reported that the XPS spectrum of Ni 2p had four characteristic peaks at 855.4, 858.7, 866.9, and 875.5 eV, which can be ascribed to Ni 2p_3/2_ and Ni 2p_1/2_ of Ni^2+^ in Ni-O, and their corresponding satellites, respectively^75^. After the adsorption of CR dye, the binding energies were shifted to 852.8 eV, 857.3 eV, 866.0 eV, and 874.8 eV (Fig. 12c). The Mg spectrum was reported to have a 1s signal at 1303.3 eV^76^. The signal of Mg 1s was shifted to 1304.1 eV after adsorption of CR dye, Fig. 12d. The two peaks of Al 2p_3/2_ and Al 2p_1/2_ corresponded to Al-O at 67.4 eV and 73.8 eV pointed by.

Plyuto et al.^77^ were displayed at 67.1 eV and 73.2 eV after adsorption of the dye molecules by thermally treated Mg/Ni/Al-LDH, Fig. 12e. The corresponding O 1s XPS spectrum was reported as four peaks at 530.2, 532.8, and 531.7 and describing the oxygen in Mg-O, Al-O, and Ni-O, respectively^78^. After the adsorption of CR dye, the locations of the peaks were shifted to 530.8 eV, 352.7 eV, 532.0 eV, and 351.1 eV as represented in Fig. 12f. The presence of adsorbed water molecules was proved by the appearance of the peak at 535.5 eV. The N 1s XPS spectrum represented two peaks at 401.8 eV and 402.3 eV correspond to N-H and N-azo-groups of CR dye molecules, (Fig. 12g) indicating the interaction between the dye molecules and the thermally treated Mg/Ni/Al-LDH^79^. The XPS spectroscopy described the interaction between the dye molecules and the prepared thermally treated Mg/Ni/Al-LDH. The observed shift in the different binding energies of the presented elements than the reported binding energies of Mg, Ni, Al, and O in the LDH compounds indicates the different interactions described while explaining the adsorption mechanism.

**Fig. 13:**
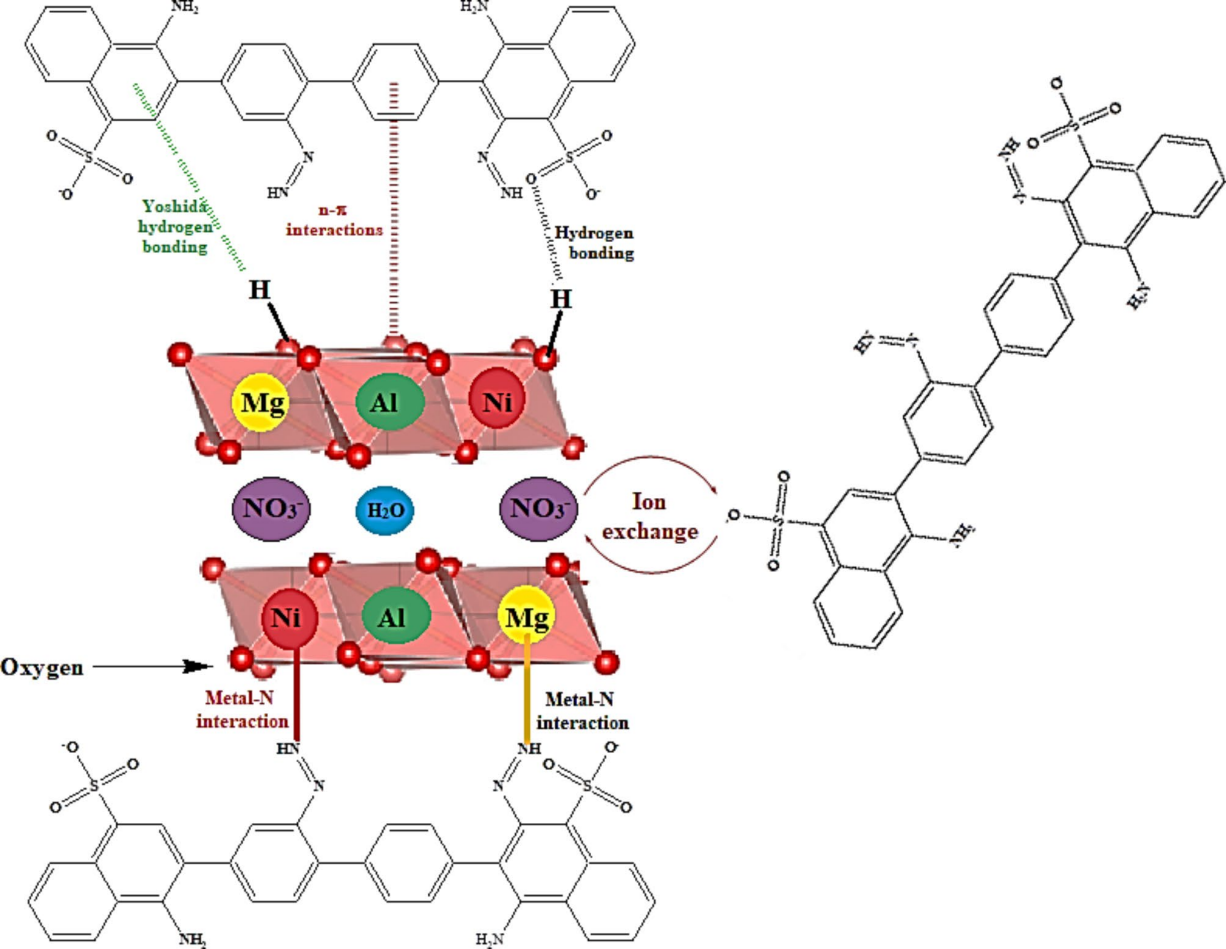
Mechanism of interaction between CR dye molecules and the thermally treated Mg/Ni/Al-LDH compound.

Figure 13 illustrates the suggested mechanism of the interaction that occurred between the Congo red dye and the prepared compounds.

### Comparison of Mg/Ni/Al-LDH and dye adsorbents

Various adsorbents used for the uptake of CR from the aqueous medium were reported.

Table 4 displayed the maximum adsorption capacity of CR using some materials from the Literature. When comparing the current study (Mg/Ni/Al-LDH and thermal treated Mg/Ni/Al-LDH) with previously reported adsorbents, it is observed the current study has the effective and potential adsorbent with high adsorption capacity for uptake of CR from aqueous solution. Comparison between the adsorption efficiency of the thermally treated Mg/Ni/Al-LDH adsorbent and that without Ni in its structure revealed that Mg/Ni/Al-LDH has an adsorption efficiency of 92.3%^80^, while the obtained efficiencies of thermally treated Mg/Ni/Al-LDH adsorbent was 97%. This finding showed the role of Ni cations in enhancing the adsorption activity of the Mg/Ni/Al-LDH. This outcome has recommended the possibilities for practical applications of Mg/Ni/Al-LDH and thermal-treated Mg/Ni/Al-LDH on an industrial scale as real alternative adsorbents.

**Table 4:** Comparison between the prepared adsorbents and the different adsorbents (reported) during the removal of Congo red dye.

Adsorbents	Contact time (min)	Adsorbent dose (g/L)	Adsorption capacity (mg/g)	References
Ni/Fe LDH	100	–	8.7959	^47^
Mg/Fe LDH	100	–	7.612	^47^
MNPs@NiFe LDH	60	0.02	79.60	^81^
MgAl–LDH	120	0.05	769.23	^82^
MgNiCo LDH	150	0.1	1194.7	^83^
Ni/Al-CO_3_ layered double hydroxide	10	–	0.9589	^84^
Mg-Fe-Al-LDH	5	–	14.75	^85^
Activated red mud	90	8	7.08	^86^
Coir pith-activated carbon	40	2	6.72	^87^
Mg/Ni/Al-LDH	100	2	15.71	This study
Thermal treated Mg/Ni/Al-LDH	100	2	18.19	

## Conclusions

The Mg/Ni/Al-LDH and thermally treated Mg/Ni/Al-LDH compounds were synthesized, characterized, and applied for the uptake of Congo red dye from industrial wastewater of textile industrial factories. The uptake process efficiency was determined and their optimum uptake efficiencies were (15.71, 18.18 mg/g) for Mg/Ni/Al-LDH and thermally treated Mg/Ni/Al-LDH compound (84%, 97%). The adsorption process followed the Freundlich adsorption isotherm according to the pseudo-second-order model. The thermal treatment of Mg/Ni/Al-LDH improved its efficiency due to the increase in the surface area, the creation of different pore types, and increasing the basicity of the produced compound after converting most of the hydroxide form into metal oxide forms. The adsorption mechanism for CR dye was administered by physisorption, electrostatic interaction, and molecular intercalation, pore filling process, hydrogen bonding, complexation, and coordination. The loaded Mg/Ni/Al-LDH and thermally treated Mg/Ni/Al-LDH by CR dye afforded high stability after acid leaching followed by base activation as the reusability ranged between 91% and 95% at the 1st cycle and reached 81% and 83% after the 3rd cycle after treating a 200 mg/L CR dye solution, respectively. Additionally, excellent CR dye removal efficiency for three real samples of tap, sea, and wastewater matrices onto thermally treated Mg/Ni/Al-LDH compounds at 98%, 88.6%, and 95% (50 mg/L dye concentrations), 92.1%, 87.9%, and 92.1% (100 mg/L dye concentrations) and 90.3%, 85.3%, and 89.7% (150 mg/L dye concentrations), respectively. Thermally treated Mg/Ni/Al-LDH compound could be regarded as a potential and promising adsorbent for polluted industrial wastewater by CR dye.

## Data Availability

The authors declare that the data supporting the findings of this study are available within the paper. Should any raw data files be needed in another format, they are available from the corresponding author upon reasonable request.
